# Gitelman Syndrome and Hypertension: A Case Report

**DOI:** 10.7759/cureus.44590

**Published:** 2023-09-02

**Authors:** Hiba Shaukat, Shazaan Nadeem, FNU Abdullah, Muhammad Muntazir Mehdi Khan, Syed W Rizvi

**Affiliations:** 1 Medicine, Allama Iqbal Medical College, Lahore, PAK; 2 Internal Medicine, Medical University Sofia, Sofia, BGR; 3 Internal Medicine, Jinnah Sindh Medical University, Karachi, PAK; 4 Surgery, Ohio State University Wexner Medical Center, Columbus, USA; 5 Medicine, University of Medicine and Dentistry of New Jersey, Newark, USA

**Keywords:** hypomagnesemia, hypochloremia, hyperreninemia, salt-losing tubulopathy, persistent hypokalemia, gitelman syndrome

## Abstract

In a patient with persistent hypokalemia, it is important to consider Gitelman syndrome, a rare, salt-wasting tubulopathy inherited in an autosomal recessive pattern. Gitelman syndrome leads to electrolyte abnormalities like hypokalemia, hypomagnesemia, and metabolic alkalosis. Typical clinical features include muscle cramps, fatigue, polydipsia, and salt cravings. Our case involves a female patient in her early 40s who visited the endocrinology clinic with symptoms of polyuria, constipation, muscle weakness, and fatigue. Electrolyte abnormalities included hypokalemia, hypomagnesemia, hypochloremia, and hyperreninemia. Initial tests, such as renal function tests, renal ultrasound, and CT scan, yielded normal results. Differential diagnosis of Gitelman syndrome and Bartter syndrome was considered due to the mutual electrolyte abnormalities of hypokalemia and metabolic alkalosis. Bartter syndrome was ruled out in our patient due to the presence of hypomagnesemia, which indicates a different defective receptor. Ultimately, genetic testing would be necessary to confirm the diagnosis of Gitelman syndrome considering the characteristic electrolyte disturbances and classic clinical presentation of fatigue, weakness, and salt craving.

## Introduction

Gitelman syndrome has a prevalence of 1 per 40,000 people. Salt-wasting tubulopathies include Gitelman syndrome and Bartter syndrome, which are inherited disorders causing salt depletion in patients [1,2]. Gitelman syndrome is an autosomal recessive tubular disorder that results from mutations in the genes for various carriers, including sodium, magnesium, and chloride [3]. In Gitelman syndrome, the defect is located in the distal convoluted tubule. This defect is usually due to a mutation in the SLC12A3 gene encoding the thiazide-sensitive NaCl cotransporter [4]. This results in defective reabsorption of ions across the tubule, resulting in electrolyte abnormalities and subsequent symptoms of the disorder. Gitelman syndrome often presents later in life, as the symptoms are usually not very severe [2]. Patients commonly present with persistent hypokalemia, metabolic alkalosis, and normal or low blood pressure. Our case report presents a patient with symptoms and electrolyte abnormalities typical of Gitelman syndrome.

## Case presentation

A 41-year-old female patient presented to the endocrinology clinic with complaints of fatigue, decreased appetite, polydipsia, polyuria, and constipation. She also complained of decreased sex drive, irritability, headaches, dizziness, muscle weakness, and wrist pain. The patient had a long history of hypertension. She was prescribed multiple antihypertensive drugs and a low-salt diet to help control her blood pressure. The patient had no significant findings on general physical exam, abdominal exam, and cardiac examination. Subsequent lab work showed low potassium, high renin, and high aldosterone (Table 1). Epinephrine and metanephrine levels were normal.

**Table 1:** Summary of laboratory test results BUN: Blood urea nitrogen; eGFR: estimated glomerular filtration rate

Lab	Lab value	Normal values
Aldosterone	50.6 ng/dL	3.1-35.4 ng/dL
Plasma Renin Activity	14.025 ng/mL/hr	0.6-4.3 ng/mL/hr
Potassium	3.1 mmol/liter	3.4-5.0 mmol/liter
Sodium	137 mmol/liter	135-145 mmol/liter
Chloride	95 mmol/liter	95-108 mmol/liter
Creatinine	0.95 mg/dL	0.8-1.3 mg/dL
BUN	13 mg/dL	8-25 mg/dL
eGFR	77 mL/min/1.73 m^2^	90 to 120 mL/min/1.73 m^2^
Calcium	9.5 mg/dL	8.5-10.5 mg/dL
Magnesium	2.4 mg/dL	1.8-2.6 mg/dL

Imaging was also done to rule out any renin-producing tumors. CT scan abdomen and ultrasound of the kidneys did not show any mass or renal artery stenosis. The patient was previously prescribed antihypertensive medications including amlodipine 2.5 mg, chlorothiazide 25 mg, and metoprolol succinate 50 mg to control her blood pressure along with potassium and magnesium supplements. Despite being on medications, the patient's blood pressure remained elevated. As a result, Lasix 20 mg was initiated, and subsequent readings indicated a return to normal blood pressure levels. Lasix and chlorothiazide were recently stopped to counter persistent hypokalemia but potassium levels still remained low while renin levels remained high. The amlodipine dose was increased to 10 mg. Potassium and magnesium supplements were continued. Follow-up visits at the clinic showed improvement in renin, aldosterone, and potassium levels. Her blood pressure was also within the normal range.

## Discussion

Gitelman syndrome is a primary defect of the thiazide-sensitive Na-Cl cotransporter in the distal convoluted tubule [5]. It is due to a mutation of genes encoding sodium-chloride cotransporter and magnesium channels. In this salt-losing disorder, we expect electrolyte anomalies such as hypokalemic metabolic alkalosis, hypomagnesemia, and hypocalciuria. The patient also might have low to normal blood pressure [6,7]. Diagnosis of such patients is usually made in adulthood as compared to other renal salt-losing tubulopathies [8]. Treatment goals usually include salt replacement and fluid replacements.

Our patient presented with a history of malaise, decreased appetite, disturbed sleep, and irritable mood, all prominent symptoms of Gitelman syndrome. She has had chronic fatigue with muscle weakness, decreased sexual drive, and irregular periods. Mild polydipsia and severe polyuria were also notable in our patient. Unlike most patients with GS, our patient presented with increased blood pressure a few months ago. Hypertension has been reported in some patients with Gitelman syndrome with increasing age due to persistent secondary hyperaldosteronism, and this could be the cause of hypertension in our patient [9].

The pathogenesis in Gitelman syndrome involves mutations in the Na-Cl cotransporter, which is the site of action of thiazide diuretics. The consequence of these electrolyte anomalies is upregulation of RAAS (renin angiotensin aldosterone system), and management involves potassium and magnesium supplementation, which our patient is taking [10,11]. The stimulation of renin and aldosterone due to the loss of salt and water leads to a compensatory rise in sodium reabsorption and passive calcium reabsorption in proximal convoluted tubules, which may be the cause of hypocalciuria.

The typical clinical presentation in Gitelman syndrome is milder than in Bartter syndrome; muscle cramps, lethargy, salt cravings, polydipsia, and polyuria. Severe cases may be seen in children less than six years of age with chondrocalcinosis, stunted growth, seizures, tetany, rhabdomyolysis, as well as ventricular arrhythmias [12,13]. In addition to clinical history, biochemical and genetic evaluation is necessary for diagnosing Gitelman syndrome. Our patient presented with the pathognomonic features of muscular weakness, fatigue, and cramps but salt cravings were absent. The literature review has shown that the presentation of Gitelman syndrome can vary widely, although our patient presented with most of the typical symptoms [14]. Most patients present in childhood or early adulthood but late presentations, as in our patient, can occur.

Our patient’s labs showed hypokalemia, hypomagnesemia, hyperreninemia, and hyperaldosteronism typical of Gitelman syndrome. Hypomagnesemia subsequently improved with magnesium supplements. In the setting of hypokalemia, differential diagnoses included Bartter syndrome. In Bartter’s syndrome, the defect is in Na-Cl reabsorption in the thick ascending limb of Henle leading to hypercalciuria [15]. Diuretic abuse was ruled out by a trial of stopping diuretic treatment, which did not result in an improvement in electrolyte levels. Primary hyperaldosteronism was ruled out by high renin levels. The patient did not report any excessive vomiting that could be the cause of the electrolyte disturbance. Ultimately, genetic testing would be required to confirm the diagnosis but is not always possible due to financial limitations [16].

## Conclusions

Our case report describes a case of Gitelman syndrome presenting in adulthood with fatigue, muscle weakness, constipation, mood disturbances, and decreased sex drive. Due to its relatively sparse incidence and lack of clinical familiarity, Gitleman syndrome can be easily misdiagnosed, leading to delayed or inappropriate treatment. Unlike most cases, our patient presented with hypertension. We emphasize the significance of considering this diagnosis in patients with typical electrolyte abnormalities and varied clinical presentations. Lab results showed persistent hypokalemia and hypomagnesemia with elevated renin and aldosterone typical of Gitelman syndrome. Renal workup showed no abnormalities of structure. The patient is being managed with potassium and magnesium supplements to alleviate symptoms and antihypertensives to control blood pressure.
